# Arthroscopic assisted versus open non-vascularized bone grafting in delayed union and nonunion of the scaphoid: a systematic review and meta-analysis

**DOI:** 10.1186/s12891-024-07723-4

**Published:** 2024-08-01

**Authors:** Atefeh Noori, Jonathan Persitz, Shawn Khan, Andrea Chan, Ryan Paul

**Affiliations:** 1 Hand Program, Division of Plastic, Reconstructive and Aesthetic Surgery, University Health Network, Toronto Western Hospital, Affiliated with University of Toronto, East Wing, 2nd Floor, Rm. 400, Toronto, Canada; 2 Division of Orthopaedic Surgery, The Hospital for Sick Children, Toronto, Canada

**Keywords:** Arthroscopic-assisted, Non-vascularized bone grafting, Scaphoid delayed union, Nonunion, Systematic review, Meta-analysis

## Abstract

**Background:**

Delayed union and nonunion of the scaphoid is a common complication often requiring surgical reconstruction and bone grafting. Our goal was to systematically assess the healing time and clinical outcomes following arthroscopic-assisted versus open non-vascularized bone grafting of the scaphoid.

**Methods:**

A comprehensive search of the MEDLINE, Embase, CINAHL (Cumulative Index to Nursing and Allied Health Literature), and Cochrane Central databases was completed from inception to September 2023. We included randomized trials and observational studies that reported outcomes following scaphoid delayed union/nonunion comparing arthroscopic-assisted vs. open non-vascularized bone grafting. Two reviewers independently extracted data and assessed the risk of bias. One investigator assessed certainty of evidence and a senior investigator confirmed the assessment. We pooled effects using random-effects models, when possible, for all outcomes reported by more than 1 study.

**Results:**

Overall, 26 studies and 822 patients were included in the study. Very low certainty evidence demonstrated that arthroscopic-assisted surgery may decrease healing time compared to open surgery (weighted mean difference [WMD] -7.8 weeks; 95%CI -12.8 to -2.8). Arthroscopic bone grafting did not result in an improvement in union rate (relative risk 1.01; 95%CI 0.9 to 1.09). The pooled data in arthroscopic graft group showed mean time to union of 11.4 weeks (95%CI: 10.4 to 12.5) with union rate of 95% (95%CI 91–98%). A single comparative study reported very low certainty evidence that arthroscopy-assisted vs. open surgery may not have an effect on pain relief (MD 0 cm, 95%CI -0.4 to 0.5 on VAS 10 cm for pain) or improving function (MD -1.2, 95% CI -4.8 to 2.3 on 100 points DASH).

**Conclusion and future directions:**

Our results suggest that arthroscopic-assisted non-vascularized bone grafting may be associated with improved average weeks to heal in comparison with open surgery for scaphoid delayed union/nonunion reconstruction with overall comparable union rates. There is insufficient evidence to assess the effects of arthroscopic-assisted reconstruction on union rate, time to union, and patient-reported outcomes in patients with other important nonunion characteristics such as established humpback deformity.

**Supplementary Information:**

The online version contains supplementary material available at 10.1186/s12891-024-07723-4.

## Introduction

Scaphoid fractures are the most common carpal fracture, typically occurring in young, active individuals following high-energy trauma or falls onto an outstretched hand [1]. While many scaphoid fractures heal uneventfully with nonsurgical management, 5 to 10% of patients may develop delayed union and/or nonunion [2, 3]. Delayed union refers to a condition where healing not achieved within 3 to 6 months after injury and nonunion is defined as no healing beyond 6 months following injury [4]. Previous studies have consistently demonstrated that earlier treatment of delayed unions/nonunions can result in great rate of successful healing [5]. A persistent scaphoid nonunion, however, can lead to Scaphoid Nonunion Advanced Collapse (SNAC) and/or notable impairment in wrist function [6].

Although many options exist for scaphoid reconstruction for nonunion, the current gold standard in surgical management of scaphoid delayed union/nonunion involves open reduction and internal fixation with non-vascularized bone grafting [7]. A previous meta-analysis suggests that screw fixation coupled with non-vascularized bone graft results in a 90% union rate [8].

Arthroscopic-assisted techniques for scaphoid bone grafting and fixation presents an alternative to conventional open grafting with the potential advantages of minimizing surgical trauma and wrist scarring while preserving the integrity of capsuloligamentous structures and the native blood supply [9–12]. Despite the utility and increased use of arthroscopic-assisted techniques, its effects compared with open surgery remains uncertain.

The aim of this study is to assess the healing time and clinical outcomes following the arthroscopic-assisted scaphoid fixation compared to open reduction internal fixation with non-vascularized bone grafting for scaphoid delayed union and nonunion.

## Methods

We followed standards for Meta-Analysis of Observational Studies [13] and PRISMA guideline for Preferred Reporting Items for Systematic Reviews and Meta-Analyses [14].

### Eligibility criteria

We included randomized control trial (RCT) and observational studies that explored the effects of arthroscopic-assisted vs. open non-vascularized bone grafting among patients with scaphoid delayed union/nonunion. We also included one-arm studies that assessed union time/rate or other clinical outcomes among patients who underwent arthroscopic-assisted graft surgery. Scaphoid delayed union was defined as a fracture with no evidence of healing within 3 to 6 months after injury, whereas nonunion refers to a scaphoid fracture that remains nonunited greater than 6 months following injury [4]. We excluded studies with acute scaphoid fractures, those associated with concomitant fractures/injuries or with history of previous scaphoid nonunion surgery, open fractures, fractures associated with wound infection, studies included vascularized bone graft, and studies with no graft. We also excluded case series (less than 10 patients), conference abstracts, thesis, protocol, and ongoing trials (or registered trial but without results).

### Literature search and study selection

We searched the MEDLINE, Embase, CINAHL (Cumulative Index to Nursing and Allied Health Literature), and Cochrane Central database from inception to September 2023 with no restriction on language of publication (Appendix A). We also searched the reference lists of all eligible studies and related systematic reviews for additional eligible studies. A pair of reviewers independently, and in duplicate, screened title/abstract and full text in online software COVIDENCE [15]. Discrepancies were resolved by discussion with senior authors.

### Data collection and risk of bias assessment

Two reviewers independently and in duplicate abstracted data from each eligible study, including the study and patients’ characteristics, the details of the surgery, graft type, fixation method, union chronicity, method of union confirmation, and the duration of follow up. Some studies included patients with proximal pole fractures. These studies were divided into three subgroups on the basis of the presence/absence of a proximal pole fracture: (1) no proximal fractures included, (2) inclusion of only proximal fractures, (3) and inclusion of patients with both of proximal and other fracture types (i.e. distal and waist). Studies were also subdivided into two subgroups based on the presence or absence of avascular necrosis (AVN). Our primary outcomes were mean time to union (continuous) and union rate (binary). We also assessed two patient-reported outcomes including: the visual analog scale (VAS) for pain, and the Disabilities of the Arm, Shoulder and Hand (DASH) for function. We also captured complications reported by the included studies. The pair of reviewers used the criteria suggested by the CLARITY group [16] to assess the risk of bias of observational studies (Supplement Tables 1 and 2) including selection bias, confidence that all patients had the condition of interest, control for confounding variables, validity of outcome assessment(s), and infrequent missing data (< 20%).

**Table 1:** Characteristics of the included studies

Author-Year	Country	Study design	Female %	No. of participants	Mean Age, yr (range or SD)	Mean follow-up in month	Operation time in minutes (range or SD)	Mean duration of nonunion in month (range or SD)	No. of delayed union/nonunion
Burnier-2023 [22]	France	One-arm observational	6	77	24 (18 to 55)	6	NR	34.8 (6 to 180)	All nonunion
Cheng-2023 [23]	China	One-arm observational	20	10	29.2 (NR)	14	NR	15 (4 to 84)	All nonunion
Chu-2011 [24]	Taiwan	One-arm observational	13	15	31 (20 to 45)	33	NR	6.5 (NR)	All nonunion
Cifras-2019 [25]	Chile	One-arm observational	0	11	23.4 (4.4)	6	NR	18.6 (16.2)	All nonunion
Cognet-2017 [26]	France	One-arm observational	13	23	26 (17 to 63)	17.3	56 (NR)	17 (6 to 60)	All nonunion
De Bie-2022 [27]	France	One-arm observational	9	47	26.3 (9.9)	17.8	NR	17.8 (17.5)	All nonunion
Delgado-Serrano-2017 [28]	Spain	One-arm observational	15	13	26 (18 to 45)	16.8	NR	14 (6 to 48)	All nonunion
Ecker-2022 [29]	Australia	One-arm observational	3	30	23 (17 to 37)	3	NR	NR	All nonunion
Gvozdenovic-2020^€^ [43]	Denmark	Retrospective cohort	13	16	30.5 (27.1)	7	140 (96 to 197) 36 (10 to 53)^¥^	27.3 (3–180) 2.5 [2–4] ^¥^	8/0; 4/4^¥^
Gvozdenovic-2021 [44]^€^	Denmark	Retrospective cohort	7	57	29 (39.9)	11	NR	16 (2 to 180)	4/4; 21/28^¥^
Gvozdenovic-2023 [45]^€^	Denmark	Retrospective cohort	7	54	28 (34.1)	24	NR	17 (2 to 15 years) 16 (2 to 10) ^¥^	9/18; 15/12^¥^
Hsiung-2021 [30]	Taiwan	One-arm observational	5	42	29 (17 to 47)	38.1	NR	11.2 (2 to 60)	All nonunion
Kang-2016 [31]	Korea	One-arm observational	2	46	28.9 (9.2)	24	NR	24.2 (11.7)	All nonunion
Kim-2015 [32]	Korea	One-arm observational	11	36	28 (11.5)	24	NR	51 (78.3)	All nonunion
Lee-2018 [33]	Korea	One-arm observational	4	27	35 (15 to 61)	18	NR	45 (NR)	All nonunion
Lee-2022 [34]	Korea	One-arm observational	7	15	44 (28 to 61)	12	177 (88 to 270)	17 (12 to 40)	All nonunion
Lin-2023 [35]	Taiwan	One-arm observational	32	34	37 (16 to 69)	20	NR	8.3 (3 to 30)	All nonunion
Liu-2019 [36]	China	One-arm observational	14	14	22 (15 to 35)	26	NR	17 (8 to 24)	All nonunion
Löw-2022 [37]	Germany	One-arm observational	24	17	34 (18 to 73)	14	77 (55 to 93)	18 (3 to 84)	All nonunion
Taek Oh-2018 [46]	Korea	Retrospective cohort	3	62	29.7 (16 to 58)	39	98 (15.4) 72.9 (14.4) ^¥^	16.1 (7.4)	All nonunion
Shih-2023 [38]	Taiwan	One-arm observational	NR	44	NR	31	NR	8.5 (4 to 24)	All nonunion
Slade-2008 [39]	US	One-arm observational	9	234	24 (15 to 59)	12	NR	20 (NR)	All nonunion
Waitayawinyu-2022 [40]^€^	Thailand	One-arm observational	9	22	34.1 (17 to 66)	32	NR	26.3 (3 to 240)	4/18
Wang-2020 [41]	Taiwan	One-arm observational	71	21	31.8 (19 to 55)	31	NR	8.6 (10.7)	All nonunion
Wu-2022 [42]^€^	China	One-arm observational	5	20	26 (13 to 40)	31	118 (90 to 150)	27 (7 to 120)	15/5
Yin-2020 [17]	China	RCT^*^	6	16	31.5 (8.4)	6	81.7 (16.6)	60 (68.4)	All nonunion

^*^Included as a single arm study as both control and intervention groups underwent the arthroscopy-assisted bone graft surgery

^¥^ first group in arthroscopic-assisted, second group in open-surgery

^€^The cases of delayed union (i.e. healing not achieved within 3 to 6 months after injury) also included

**Table 2:** GRADE evidence profile: arthroscopic versus open non-vascularized bone grafting in patients with delayed union and nonunion of the scaphoid

# of studies	# of patients	Risk of bias	inconsistency	indirectness	imprecision	Publication bias	Treatment effect (95%CI)	Overall certainty of evidence
**Mean time to union (weeks)**								
3 retrospective cohort studies [43–45]	110 (intervention: 39, control: 71)	Serious risk of bias	Not serious	Not serious	Not serious	Not detected	WMD − 7.8 weeks (-12.8 to -2.8)	Very low
**Overall mean time to union in arthroscopic-assisted group**								
14 single-arm studies [22–25, 28, 32–36, 38, 40–42]	338	Serious risk of bias	Serious inconsistency (visual inspection)	Not serious	Not serious	Not detected	11.4 weeks (10.4 to 12.5)	Very low
**Union rate (%)**								
4 retrospective cohort studies [43–46]	189 (intervention: 71, control: 118)	Serious risk of bias	Not serious	Not serious	Serious	Not detected	RR 1.01 (0.9 to 1.0) In arthroscopic-assisted: 94% (86–99%) In open bone-grafting: 90% (81–96%)	Very low
**Overall union rate in arthroscopic-assisted group**								
21 single-arm studies [17, 22–42]	675	Serious risk of bias	Not serious	Not serious	Not serious	Not detected	95% (91–98%).	Very low
**Pain relief**								
1 retrospective [46] cohort	62 (intervention: 28, control: 34)	Serious risk of bias	-	-	Serious	-	MD 0 cm (-0.4 to 0.5 on 10 cm VAS)	Very low
**Physical function**								
1 retrospective [46] cohort	62 (intervention: 28, control: 34)	Serious risk of bias	-	-	Serious	-	MD -1.2 point (-4.8 to 2.3 on 100 points DASH)	Very low

Weighted mean difference: WMD; Relative risk: RR; visual analog scale: VAS

### Data analysis

We conducted the meta-analysis for single-armed and studies with control group separately. The RCT [17] was included as single-arm study as both arms received the intervention (arthroscopic-assisted bone graft fixation). We also reported the results of studies that were not possible to pool. For continuous outcomes that were reported by more than two studies, we pooled data and calculated weighted mean difference (WMD) with associated 95% confidence interval (CI) reported. For the union rate as a binary outcome, we calculated relative risk (RR) along with 95% CI. We conducted all meta-analyses with random-effects models and the DerSimonian-Laird method [18]. We pooled data from single-arm studies to calculate the overall mean time to union and percentage of union amongst the arthroscopic-assisted bone graft group. For studies that reported continuous outcomes (i.e. mean time to union and patient-reported outcomes) as median and IQR, we derived the mean and SD using the method presented by Wan et al. [19]. We used Stata (StataCorp, Release V.15.1) for all analyses. Comparisons were two tailed using a threshold of *p* ≤ 0.05.

### Subgroup and sensitivity analysis

When we had at least two studies in each subgroup, we explored the source of heterogeneity by assessing the five pre-specified subgroups, assuming shorter healing time with studies that: (1) excluded proximal pole fractures, (2) excluded AVN, (3) included patients with shorter duration of nonunion (i.e. defined less than 18 months), (4) studies which used iliac crest graft site vs. other graft sites, and (5) studies which used screw fixation vs. other types of fixation. We planned to explore the publication bias when there were at least 10 studies for meta-analysis.

### Certainty of evidence

One reviewer used the Grading of Recommendations Assessment, Development and Evaluation (GRADE) [20] approach to assess the certainty of evidence for each outcome as high, moderate, low or very low and confirmed the results with the senior author. The domains of risk of bias, imprecision, inconsistency, indirectness, and publication bias were assessed. We rated down for imprecision if the 95% CI associated with pooled outcomes included no effect (i.e. zero for continuous outcomes and one for binary outcome). For the inconsistency domain, we evaluated the I^2^ statistic and visual inspection of forest plot for pooled outcomes [21].

## Results

**Fig. 1:**
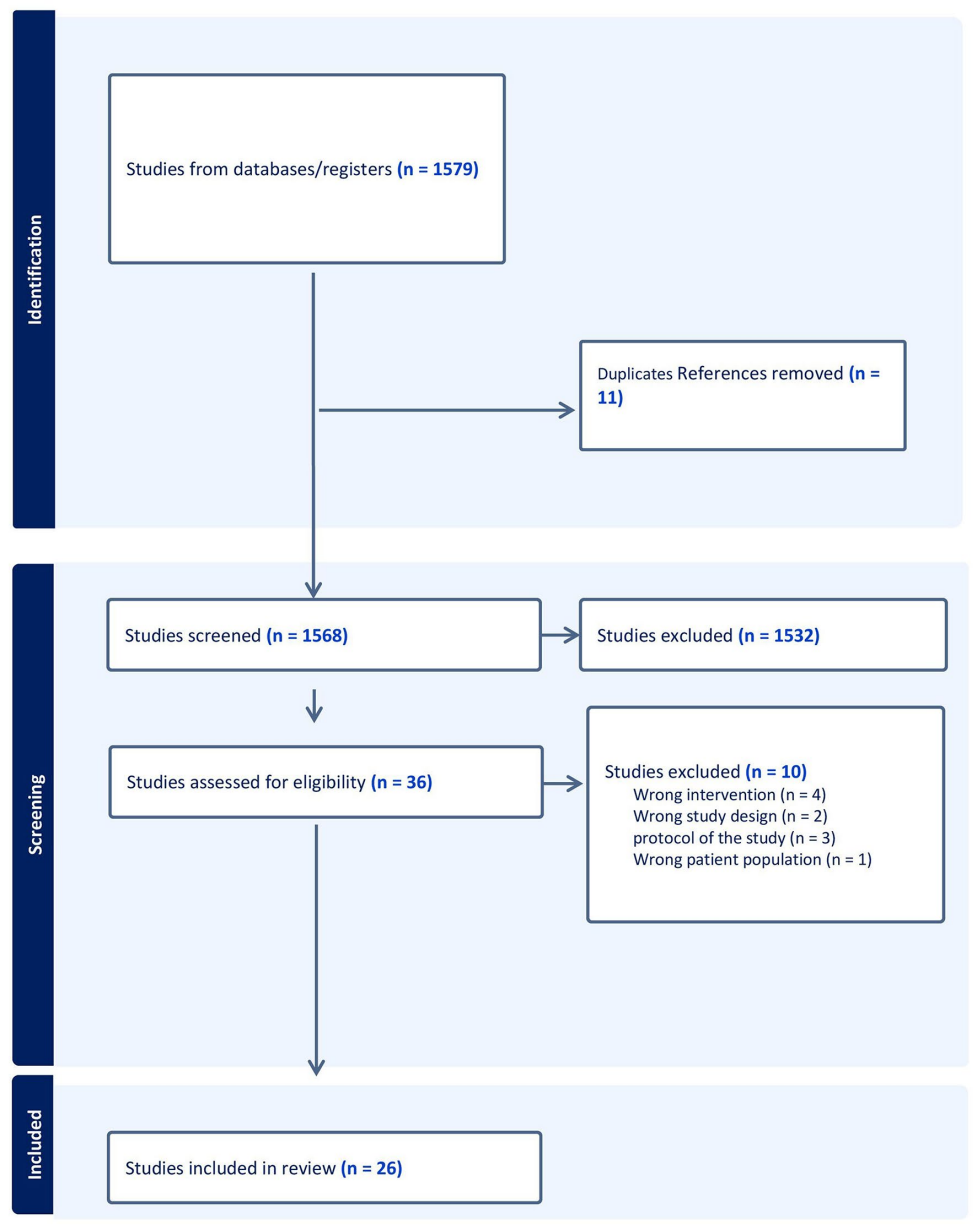
Preferred reporting items for systematic review and meta-analysis flow chart

Of 1568 records identified, we reviewed 36 articles in full text, and 26 studies with 822 participants were eligible and included in our review (Fig. 1). There were 21 observational single arm studies [22–42], four retrospective cohort studies [43–46], and one RCT [17]. All studies enrolled patients with scaphoid nonunion or a combination of nonunion and delayed union [40, 42–45]. The retrospective cohort studies compared arthroscopic-assisted bone graft reconstruction surgery versus open surgery. The included RCT [17], recruited patients with scaphoid nonunion without displacement or with minimal displacement, and compared arthroscopic-assisted fixation and bone grafting with 3D guided system versus without the 3D guided system. We included this RCT as a single arm study as both control and intervention groups underwent arthroscopic-assisted surgery. One retrospective cohort study was excluded because authors used salvage technique in patients with scapholunate advanced collapse (SNAC) [47]. Another one-armed study also excluded as they included cases with previous history of scaphoid fracture who underwent the nonunion surgery [48]. Amongst all included studies, the percentage of female individuals was 18% and participants’ age ranged from 22 to 44 years old. The duration of nonunion ranged from 6 to 60 months and participant follow up ranged from 3 to 39 months (Table 1).

Regarding the graft site, 10 included studies used distal radius [22, 23, 25–27, 30, 32, 35, 37, 39], and 16 studies used either iliac crest [17, 28, 29, 31, 33, 34, 42, 46] or combination of iliac crest and distal radius or olecranon [24, 36, 38, 40, 41, 43–45]. All studies reporting arthroscopic-assisted surgery harvested the autologous cancellous bone grafts from either the distal radius or the iliac crest, depending on the size of the nonunion gap. Only one included study injected a bone graft substitute that was packed with increasing firmness into the nonunion site and proximal pole [24]. Most of the included studies [22, 25–28, 36, 38, 39, 41, 42, 44, 46] used a combination of fixation method (i.e. headless compression screw and K-wire), eight studies used headless compression screw [23, 24, 31, 35, 37, 40, 43, 45], and six of them used K-wire method [17, 29, 30, 32–34]. Three of the studies included patients with stage I SNAC [33, 34, 41]. One study also excluded scaphoid nonunion patients without coexisting ligament injuries [31].

### Risk of bias of included studies

All observational studies with control group were at high risk of bias (Supplement Table 3). The most common limitation in these studies was due to inadequate adjustment for potential confounders. The majority of the single-armed studies also were at high risk of bias (20 out of 22) due mostly to non-representative samples (Supplement Table 4).

### Outcomes

#### Mean time to union in weeks

Very low certainty evidence from three studies [43–45] suggest that arthroscopy-assisted bone grafting and fixation may decrease mean time to healing by 7.8 weeks (95%CI -12.8 to -2.8 weeks) compared to open surgery (Table 2, Supplement Fig. 1).

The overall pooled mean time to union following arthroscopic-assisted bone grafting surgery and fixation for delayed union/nonunion scaphoid was 11.4 weeks (95%CI 10.4 to 12.5 weeks; Table 2, Supplement Fig. 2). The mean time to union did not differ based on the chronicity of the nonunion (Supplement Fig. 3, a test of interaction *p* = 0.36) or the proximal pole fracture location (Supplement Fig. 4, a test of interaction *p* = 0.88).

#### Union rate

From the four observational studies [43–46], arthroscopic-assisted bone grafting and fixation did not result in a significant improvement in union rate compared with open surgery (RR 1.01; 95%CI 0.9 to 1.09; very low certainty evidence; Table 2, Supplement Fig. 5). The overall pooled union rate from 21 single-arm studies [17, 22–42] in arthroscopic graft group was 95% (95%CI 91–98%; Supplement Fig. 6). The result did not differ based on the subgroups of chronicity, studies that excluded proximal pole fracture and AVN, and site of the graft (Supplement Figs. 7, 8, 9, 10 and 11).

#### Pain relief and physical function

Very low certainty evidence from one study [46] showed that arthroscopic-assisted bone grafting and fixation may not be associated with improved pain (MD 0 cm; 95%CI -0.4 to 0.5 on 10 cm VAS of pain) or physical function compared with open bone grafting and fixation (MD -1.2, 95% CI: -4.8 to 2.3 on 100 points DASH; Table 2). However, in one-arm studies [22, 23, 25, 27, 28, 30–32, 35, 42, 45] conducted amongst patients who underwent arthroscopic-assisted bone grafting and fixation, it was found that post-operative pain and function substantially improved compared to their pre-surgery condition (Supplement Figs. 12 and 13).

#### Complications

Three studies reported complications as those requiring revision surgery 12,15, 29) and one study reported minor adverse events [31] (Supplement Table 5).

## Discussion

This meta-analysis demonstrated that arthroscopic-assisted reconstruction with non-vascularized bone grafting and fixation for scaphoid delayed union/nonunion may be associated with faster healing time compared to open surgery, while union rates are comparable (94% vs. 90% in arthroscopic and open surgery respectively). The pooled data of this study showed a high union rate after arthroscopic-assisted surgery which was not impacted by either chronicity, fracture location or AVN.

Our study is the first meta-analysis that has compared arthroscopic-assisted versus open reconstruction with non-vascularized bone graft and fixation for scaphoid delayed union/nonunion. We conducted a comprehensive search, pooled data for time to healing, evaluated the risk of bias among individual studies, and used GRADE approach to rate the certainty of evidence of pooled data. Our data however, is limited in its ability to further parse out the relationship between nonunion characteristics (other than chronicity and fracture site), and successful arthroscopic reconstruction with bone grafting. Specifically, the current study is unable to conclude the effect of fracture or intercarpal instability (i.e., humpback deformity or dorsal intercalated segment instability [DISI]) on arthroscopic feasibility and union rate. Amongst the included studies, arthroscopic-assisted scaphoid reconstruction was typically reserved for more recently established nonunion which had minimal resorption, little sclerosis, absent AVN, and no humpback deformity [23]. Five of the studies in this review presented results including both delayed and nonunion cases. However, the results were not reported separately based on this critical characteristic. We were therefore unable to explore the relative influence of delayed union versus non-union on our primary and secondary outcomes. Sensitivity analysis and exclusion of these studies did not considerably change the findings of the current review. However, there are certainly differences in the prognosis for fractures that may be expected to heal given enough time without intervention (delayed union) and those that will not heal without intervention (established non-unions). Importantly, subgroup analysis based on chronicity (greater or less than 18 months), which may be a reasonable surrogate for non-union, did not find a significant difference between groups. Future investigations would do well to include specific and consistent definitions and report results separately where possible to allow for appropriate subgroup analysis. Moreover, the very low certainty evidence resulted in uncertainty regarding the effect of arthroscopic-assisted versus open reconstruction surgery on healing time and rate in scaphoid delayed union/nonunion.

In terms of the residual humpback deformity correction using arthroscopic-assisted technique, two studies [27] reported the ability to achieve union and correct the DISI deformity [49]. However, the second study [49] did not use bone graft and authors noted that preoperative scaphoid imaging may not always predict the type of scaphoid that can be treated arthroscopically. Instead, they found that the presence of hemorrhagic petechiae observed arthroscopically was a primary predictive factor for successful union based on their experience. Overall, this literature shows that arthroscopy serves as a valuable adjunct for scaphoid delayed union/nonunion evaluation and serves as an viable surgical option for reconstruction in selected cases, provided that the principles of pseudoarthrosis debridement and rigid internal fixation are adhered to [50].

Our results also suggest that arthroscopic-assisted scaphoid reconstruction may have an important clinical benefit for patients by substantially improving healing time by approximately 7.8 weeks compared with open surgery. Arthroscopic-assisted reconstruction may as such, also result in decreased immobilization and time off work/sport. In addition to increased time to union, open reconstruction carries greater surgical site morbidity by disrupting the capsuloligamentous structures and potentially causing post-operative wrist stiffness and functional issues. Most included studies in this review, however, were single-armed, limiting meta-analysis and accurate conclusions on functional outcomes and details of return to work/sport after arthroscopic-assisted scaphoid reconstruction comparing with open surgery. This study was unable to identify patterns in either union rate or time to union based on the type or site of bone graft used (i.e. autologous iliac crest or distal radius, injectable bone substitute) due to lack of variability. Despite controversial literature, most studies suggested a consistent union rate irrespective of bone graft type [51]. Caloia et al. [49] reported a 91.7% union rate without the use of bone graft. These results, however, were in a highly selected range of patients of which all were young non-smokers without excessive scaphoid sclerosis or a small proximal pole fragment, and who demonstrated hemorrhagic petechiae on arthroscopic evaluation.

This meta-analysis demonstrated that the overall union rate was not statistically significantly different between the arthroscopic-assisted and open reconstruction groups, and both groups offered high rate of union. A narrative systematic review also reported that arthroscopic-assisted reconstruction had a high rate of union for the management of scaphoid nonunion [52]. Our union rate results align with those reported in other systematic reviews for open reconstruction using non-vascularized bone graft and screw fixation [12, 53]. The current study demonstrated that arthroscopic-assisted scaphoid reconstruction with bone graft achieves union with minimal complications, comparable to the gold standard open technique, particularly for highly selected types of scaphoid nonunion.

## Conclusion

Our results suggest that arthroscopic-assisted non-vascularized bone grafting may be associated with improved time to union in comparison with open surgery for scaphoid delayed union/nonunion reconstruction with overall comparable union rates. There is insufficient evidence to assess the effects of arthroscopic-assisted reconstruction on union rate, time to union, and patient-reported outcomes in patients with other important nonunion characteristics such as established humpback deformity.

### Electronic supplementary material

Below is the link to the electronic supplementary material.

Supplementary Material 1

## Data Availability

Data are available on reasonable request to the corresponding author at: ryan.paul2@uhn.ca.
