# Supplementary material for: Arthroscopic assisted versus open non-vascularized bone grafting in delayed union and nonunion of the scaphoid: a systematic review and meta-analysis
Source: BMC Musculoskelet Disord. 2024 Aug 1;25:611. doi: 10.1186/s12891-024-07723-4 (PMC11293166; doi:10.1186/s12891-024-07723-4)
Supplement: Supplementary file 1 — Supplementary Material 1 [file 12891_2024_7723_MOESM1_ESM.docx]

Supplementary Material

Arthroscopic assisted versus open non-vascularized bone grafting in delayed and nonunion of the scaphoid: a systematic review and meta-analysis

**Content:**

Contents

[Appendix A: search strategy 3](#_Toc160615263)

[Supplement Table 1: Detailed guidance for risk of bias assessment retrospective or prospective chart-reviews with control group 4](#_Toc160615264)

[Supplement Table 2: Detailed guidance for risk of bias assessment retrospective or prospective chart-reviews with no control group 8](#_Toc160615265)

[Supplement Table 3: Risk of bias assessments for chart reviews with control group 10](#_Toc160615266)

[Supplement Table 4: Risk of bias assessments for one-arm studies with no control group 11](#_Toc160615267)

***Comparing arthroscopic-assisted vs. open reconstruction surgery***

[Supplement Figure 1: forest plot for mean time to union in weeks in patients with delayed/nonunion scaphoid who received arthroscopic-assisted vs. open reconstruction surgery 13](#_Toc160615268)

***In one-arm studies among patients who underwent arthroscopic-assisted reconstruction***

[Supplement Figure 2: forest plot for mean time to union in weeks in patients with delayed/nonunion scaphoid who received arthroscopic-assisted reconstruction surgery 14](#_Toc160615269)

***Subgroup analysis***

[Supplement Figure 3: forest plot for mean time to union in weeks in patients with delayed/nonunion scaphoid who received arthroscopic-assisted reconstruction surgery by chronicity of nonunion 15](#_Toc160615270)

[Supplement Figure 4: forest plot for mean time to union in weeks in patients with delayed/nonunion scaphoid who received arthroscopic-assisted reconstruction surgery by proximal pole fracture location 16](#_Toc160615271)

***Union rate comparing arthroscopic-assisted vs. open reconstruction surgery***

[Supplement Figure 5: forest plot for risk ratio of union rate in patients with delayed/nonunion scaphoid who received arthroscopic-assisted vs. open reconstruction surgery 17](#_Toc160615272)

***Union rate in one-arm studies among patients who underwent arthroscopic-assisted reconstruction***

[Supplement Figure 6: forest plot for overall union rate in patients with delayed/nonunion scaphoid who received arthroscopic-assisted 18](#_Toc160615273)

***Subgroup analysis***

[Supplement Figure 7: forest plot for overall union rate in patients with delayed/nonunion scaphoid who received arthroscopic-assisted by chronicity of nonunion 19](#_Toc160615274)

[Supplement Figure 8: forest plot for overall union rate in patients with delayed/nonunion scaphoid who received arthroscopic-assisted reconstruction surgery by proximal pole fracture location 20](#_Toc160615275)

[Supplement Figure 9: forest plot for overall union rate in patients with delayed/nonunion scaphoid who received arthroscopic-assisted reconstruction surgery by avascular necrosis (AVN) 21](#_Toc160615276)

[Supplement Figure 10: forest plot for overall union rate in patients with delayed/nonunion scaphoid who received arthroscopic-assisted reconstruction surgery by type of fixation 22](#_Toc160615277)

[Supplement Figure 11: forest plot for overall union rate in patients with delayed/nonunion scaphoid who received arthroscopic-assisted reconstruction surgery by graft site 23](#_Toc160615278)

***Comparing the patients reported outcomes before and after arthroscopic-assisted surgery***

[Supplement Figure 12: comparing patient reported pain (based on VAS 0 to 10 cm) before and after arthroscopic-assisted reconstruction surgery in patients with delayed/nonunion scaphoid 24](#_Toc160615279)

[Supplement Figure 13: comparing patient reported function (based on DASH 0 to 100 points) before and after arthroscopic-assisted reconstruction bone graft surgery in patients with delayed/nonunion scaphoid 25](#_Toc160615280)

***Complications***

[Supplement Table 5: Complication rate reported by studies 26](#_Toc160615281)

# Appendix A: search strategy

| Database | Search Terms | Results |
| --- | --- | --- |
| Embase | 1. exp Scaphoid fracture/  2. (exp Scaphoid bone/ OR exp Carpal bone/ OR exp Wrist fracture/) adj3 (exp Fracture healing/ OR exp Fracture nonunion/ OR union.ti,ab OR SNAC.ti,ab)  3. exp Bone graft/ OR exp Bone transplantation/  4. (1 OR 2) AND 3 | 1054 |
| MEDLINE | 1. exp Scaphoid fracture/  2. (exp Scaphoid bone/ OR exp Carpal bone/ OR exp Wrist fracture/) adj3 (exp Fracture healing/ OR exp Fracture nonunion/ OR union.ti,ab OR SNAC.ti,ab)  3. exp Bone graft/ OR exp Bone transplantation/  4. (1 OR 2) AND 3 | 359 |
| CINAHL | S1. (MM "Carpal Fractures") OR "scaphoid fracture"  S2. (MM "Fracture Healing") OR (MM "Fractures, Ununited")  S3. (MM "Bone Transplantation") | 267 |
| Cochrane CENTRAL | 1. (scaphoid fracture):ti,ab,kw  2. ((scaphoid* OR carpal bone*) AND (fracture* OR nonunion)):ti,ab,kw  3. (bone graft* OR bone transplantation):ti,ab,kw  4. (1 OR 2) AND 3 | 179 |

Total results = 1859

De-duplication = 1571

#

**Risk of Bias Assessment**

# Supplement Table 1: Detailed guidance for risk of bias assessment retrospective or prospective chart-reviews with control group

| **Domain** | **Judgment** |
| --- | --- |
| 1. **Did the study match participants for all variables that are associated with the outcome of interest or did the statistical analysis adjust for these prognostic variables?** (This item queries how confident we are that the reported association or lack thereof is not due to confounding). | **Definitely yes (low risk):** studies that adjusted based on all important covariates including age, time from injury (duration of nonunion), baseline pain, fracture site, and smoking status.  **Probably yes (low risk):** studies that adjusted at a minimum for baseline pain and time from injury.  **Probably no (high risk):** studies that did not provide any details about analysis method.  **Definitely no (high risk):**  Studies that did not adjust at a minimum. |
| 1. **Was selection of exposed and non-exposed cohorts drawn from the same population?** (this item queries whether participants who underwent arthroscopically assisted bone grafting or open surgery were drawn from the same population) | **Definitely yes (low risk):** Studies in which selection for participation is not dependent on exposure status (arthroscopically assisted bone grafting).  **Probably yes (low risk):** studies that did not provide enough information about recruitment to judge whether recruitment into the study was dependent on exposure status or not.  **Probably no (high risk): NA**  **Definitely no (high risk):** studies that compared arthroscopically assisted bone grafting and open surgery from different cohort. |
| 1. **Can we be confident in the assessment of exposure (intervention)?** | **Definitely yes (low risk):** if study reported how defined the arthroscopically assisted bone grafting.  **Probably yes (low risk):** NA  **Probably no (high risk):** NA  **Definitely no (high risk):** no ascertainment that intervention was defined properly. |
| 1. **Can we be confident in the assessment of the presence or absence of prognostic factors?** | **Definitely yes (low risk):** when patients self-reported the prognostic factors.  **Probably yes (low risk):** when the method of assessment was not reported, it was considered as probably yes.  *Note that for this item, we are only concerned with the measurement of the prognostic factors that mentioned in item number 1 as minimum adjusted variables. |
| 1. **Were co-interventions similar between groups?** | **Definitely yes (low risk):** study reported that co-intervention other than study intervention were limited and post-op protocol for rehabilitation and immobilization were similar after surgery.  **Probably yes (low risk):** when co-intervention usage was approximately balanced between both intervention and control groups.  **Probably no (high risk):** when study did not provide enough information about other intervention that participants may use.  **Definitely no (high risk):** when participants were allowed to use all other co-interventions that could affect the outcome of the study such as physiotherapy. |
| 1. **Was the follow up of cohorts adequate?** (This item queries the risk of bias associated with loss to follow-up and missing outcome data). | **Definitely yes (low risk):** the retention rate was at least 90% through the study.  **Probably yes (low risk):** the retention rate approximately 80-89% and loss to follow-up unlikely to be related to the outcome.  **Probably no (high risk):** the retention rate approximately 80-89%, however its rate likely to be related to the loss to follow-up. For instance, if patients were required to come to clinic for outcome measurement, patients who had poorer outcomes, or on the other hand, patients who were feeling better, may be less likely to attend the clinic.  Loss to follow-up did not report or could not estimate.  **Definitely no (high risk):** loss to follow-up more than 20%. |
| 1. **Can we be confident in the assessment of outcome?** (This item queries our confidence in the accuracy of the measurement of the outcome). | **Definitely yes (low risk):** study used a valid method for defining the healing such as using clinically and radiographic methods, and validated/reliable measurement for pain assessment (e.g. VAS, NRS).  **Probably yes (low risk):** NA  **Probably no (high risk):** when study did not provide enough information about the outcome measurement.  **Definitely no (high risk):** study used non-validated/reliable methods. |

# Supplement Table 2: Detailed guidance for risk of bias assessment retrospective or prospective chart-reviews with no control group

| **Domain** | **Judgment** |
| --- | --- |
| **Is the source population (sampling frame) representative of the general population?** | **Definitely yes (low risk):** participants were selected from a representative sample (e.g. national population registry)  **Probably yes (low risk):** single community center, however the center was the only referral center that provided care to scaphoid nonunion patients.  **Probably no (high risk):** based on the provided information source population could not be defined.  **Definitely no (high risk):** sampling from one center or clinic or hospital or patients selected through using convenience sampling. |
| **Is the assessment of the outcome accurate both at baseline and at follow-up?** | **Definitely yes (low risk):** study used a valid method for defining the healing such as using clinically and radiographic methods, and validated/reliable measurement for pain assessment (e.g. VAS, NRS).  **Probably yes (low risk):** NA  **Probably no (high risk):** when study did not provide enough information about the outcome measurement.  **Definitely no (high risk):** used of different instruments at different follow-up intervals with concern of accuracy of responses, or used invalidated/reliable instruments. |
| **Is there little missing data?** | **Definitely yes (low risk):** the retention rate was at least 90% through the study.  **Probably yes (low risk):** the retention rate approximately 80-89% and loss to follow-up unlikely to be related to the outcome.  **Probably no (high risk):** the retention rate approximately 80-89%, however its rate likely to be related to the loss to follow-up. For instance, if patients were required to come to clinic for outcome measurement, patients who had poorer outcomes, or on the other hand, patients who were feeling better, may be less likely to attend the clinic.  Loss to follow-up did not report or could not estimate.  **Definitely no (high risk):** loss to follow-up more than 20%. |

# Supplement Table 3: Risk of bias assessments for chart reviews with control group

| Study | Were the exposed and unexposed drawn from same population? | Are we confident in the assessment of exposure? | Can we be confident in the assessment of the presence or absence of prognostic factors? | Can we be confident in the outcome assessment? | Was there adequate follow-up? | Were the co-interventions similar? | Did the authors adjust for different confounders? | Overall risk of bias |
| --- | --- | --- | --- | --- | --- | --- | --- | --- |
| Gvozdenovic et al- 2019 | Low risk | Low risk | Low risk | Low risk | Low risk | Low risk | High risk | High risk |
| Gvozdenovic et al- 2023 | High risk | Low risk | Low risk | Low risk | Low risk | Low risk | High risk | High risk |
| Gvozdenovic et al- 2021 | Low risk | Low risk | Low risk | Low risk | Low risk | Low risk | High risk | High risk |
| Taek Oh2018 | Low risk | Low risk | High risk | Low risk | Low risk | Low risk | High risk | High risk |

# Supplement Table 4: Risk of bias assessments for one-arm studies with no control group

| **Study** | Is the source population (sampling frame) representative of the general population? | Is the assessment of the outcome accurate both at baseline and at follow-up? | Is there little missing data? | Overall risk of bias |
| --- | --- | --- | --- | --- |
| Burnier2023 | Low risk | Low risk | Low risk | Low risk |
| Cheng2023 | High risk | Low risk | Low risk | High risk |
| Chu2011 | High risk | Low risk | High risk | High risk |
| Cifras2019 | High risk | Low risk | Low risk | High risk |
| Cognet2017 | High risk | Low risk | Low risk | High risk |
| De Bie2022 | High risk | Low risk | Low risk | High risk |
| Delgado-Serrano2017 | High risk | Low risk | Low risk | High risk |
| Ecker2022 | High risk | Low risk | Low risk | High risk |
| Hsiung2021 | High risk | Low risk | Low risk | High risk |
| kang2016 | High risk | Low risk | Low risk | High risk |
| kim2015 | High risk | Low risk | Low risk | High risk |
| Lee2018 | High risk | Low risk | Low risk | High risk |
| lee2022 | High risk | Low risk | Low risk | High risk |
| Lin2023 | High risk | Low risk | Low risk | High risk |
| Liu2019 | High risk | Low risk | Low risk | High risk |
| Löw2022 | High risk | Low risk | Low risk | High risk |
| Shih2023 | High risk | Low risk | High risk | High risk |
| Slade2008 | High risk | Low risk | Low risk | High risk |
| Waitayawinyu2021 | High risk | Low risk | High risk | High risk |
| Wang2020 | High risk | Low risk | High risk | High risk |
| Wu2022 | Low risk | Low risk | Low risk | Low risk |
| Yin et al-2020^¥^ | High risk | Low risk | Low risk | High risk |

^¥^ This study was an RCT, however, as both arms received the intervention (arthroscopic-assisted bone graft surgery) we included it as a single-arm study.

# Supplement Figure 1: forest plot for mean time to union in weeks in patients with delayed/nonunion scaphoid who received arthroscopic-assisted vs. open reconstruction surgery

# Supplement Figure 2: forest plot for mean time to union in weeks in patients with delayed/nonunion scaphoid who received arthroscopic-assisted reconstruction surgery

# Supplement Figure 3: forest plot for mean time to union in weeks in patients with delayed/nonunion scaphoid who received arthroscopic-assisted reconstruction surgery by chronicity of nonunion. A test of interaction p=0.36

# Supplement Figure 4: forest plot for mean time to union in weeks in patients with delayed/nonunion scaphoid who received arthroscopic-assisted reconstruction surgery by proximal pole fracture location. A test of interaction p=0.88.

# Supplement Figure 5: forest plot for risk ratio of union rate in patients with delayed/nonunion scaphoid who received arthroscopic-assisted vs. open reconstruction surgery

# Supplement Figure 6: forest plot for overall union rate in patients with delayed/nonunion scaphoid who received arthroscopic-assisted

# Supplement Figure 7: forest plot for overall union rate in patients with delayed/nonunion scaphoid who received arthroscopic-assisted by chronicity of nonunion

A test of interaction p=0.57

# Supplement Figure 8: forest plot for overall union rate in patients with delayed/nonunion scaphoid who received arthroscopic-assisted reconstruction surgery by proximal pole fracture location

A test of interaction p= 0.63

# Supplement Figure 9: forest plot for overall union rate in patients with delayed/nonunion scaphoid who received arthroscopic-assisted reconstruction surgery by avascular necrosis (AVN)

A test of interaction p= 0.41

# Supplement Figure 10: forest plot for overall union rate in patients with delayed/nonunion scaphoid who received arthroscopic-assisted reconstruction surgery by type of fixation

A test of interaction p=0.65

Combination: screw and K-wire

Supplement Figure 11: forest plot for overall union rate in patients with delayed/nonunion scaphoid who received arthroscopic-assisted reconstruction surgery by graft site. A test of interaction p=0.64

*other: combination of iliac crest and distal radius or olecranon

# Supplement Figure 12: comparing patient reported pain (based on VAS 0 to 10 cm) before and after arthroscopic-assisted reconstruction surgery in patients with delayed/nonunion scaphoid

# Supplement Figure 13: comparing patient reported function (based on DASH 0 to 100 points) before and after arthroscopic-assisted reconstruction bone graft surgery in patients with delayed/nonunion scaphoid

# Supplement Table 5: Complication rate reported by studies

| **Study** | **Rate of complication** | **Type of complication** |
| --- | --- | --- |
| Hsiung et al. 2021 | 2 out of 41 patients | Revision surgery with regional vascular bone graft. All among patients with proximal pole nonunion. |
| Lee et al. 2018 | 1 out of 27 patients | Revision surgery with an open corticocancellous bone graft. |
| Slade et al. 2008 | 13% | Revision surgery for additional bone grafting. |
| Kang et al. 2016 | 2 out of 46 patients | Minor complication: One case had superficial wound infection. One case needed Pin removal. |
